# Obituary

**Published:** 1869-02

**Authors:** 


### OBITUARY.

DIED—Dr. B. F. Mills, on Friday, Dec. 11, 1868, at the house of his brother, Dr. G. A. Mills, Brooklyn, New York. Dr. Mills had been in feeble health for several months before his death, so that it was not wholly unexpected, either by himself or his friends. He was a graduate of the Ohio College of Dental Surgery, session of 1867 and 1868. He was a young man of superior professional attainments; one for whom high hopes were entertained in a professional field. The profession, as well as his immediate friends, have lost much by his early demise.

